# To the Reader

**Published:** 1790

**Authors:** 


### TO THE READER.

THE indulgent manner in which the London Medical Journal has been received by the Public, and the numerous and valuable Communications with which the Editor has been favoured by his Correſpondents, have induced him to perſevere in bringing it out, at ſtated quarterly periods, much longer than has well ſuited his other avocations. But this mode of publication having of late been attended with great inconvenience both to his profeſſional engagements and to the deliberate management of the work, he flatters himſelf he ſhall not be ſuſpected of any diminution of zeal to ſerve the Public, if now, after having brought this Collection to the end of the tenth year, he ſhould be deſirous of conducting his future labours in a way more convenient and ſatisfactory to himſelf.

He is aware that by making ſuch an alteration in the form of the Journal as might enable him to continue it at his leiſure, he ſhould retain the advantages which an eſtabliſhed work may be expected to have over a new undertaking; but the reſpect he owes to his readers (many of whom might, perhaps, conſider any farther change in the mode of publication as too great a deviation from his original plan) induces him rather to bring the preſent work to a cona conclusion, and to begin a new Collection, the arrangement of which, so far as relates to the periods of publication, shall be better adapted to his other avocations.

The LONDON MEDICAL JOURNAL will accordingly end with the present volume; and, to complete it, a General Index is added of the contents of the work.

The Collection that is intended to succeed it will be entitled MEDICAL FACTS AND OBSERVATIONS. Its object, like that of the present work, will be to contribute to the improvement and diffusion of medical knowledge; and, like this, it will consist of original papers communicated by Correspondents, or of materials collected from the Transactions of learned Societies and from other printed works.

The Editor proposes to bring out a part of this new work as often as he shall have got together materials sufficient to form a small volume, of fifteen or sixteen sheets, in octavo; a volume of this size, as it will enable him to make the periods of publication more frequent, seeming to be better calculated for the purposes of the work than one of greater bulk.

Communications for the intended work may be addressed to DR. SIMMONS, Poland Street, London.

